# Robot-Assisted Excision of Congenital Mega-Seminal Vesicle Associated with Zinner's Syndrome

**DOI:** 10.1089/cren.2018.0047

**Published:** 2019-03-18

**Authors:** Campbell F. Bryson, Sophia Delpe, Stephanie Tatzel, Aaron Perecman, Adam Hittelman, Michael S. Leapman

**Affiliations:** Department of Urology, Yale University School of Medicine, New Haven, Connecticut.

**Keywords:** seminal vesicle cyst, Zinner's syndrome, minimally invasive, robot-assisted laparoscopic surgery

## Abstract

***Background:*** Abnormalities of mesonephric ducts are rare congenital conditions, which can present with vague symptoms in otherwise healthy men. Zinner's syndrome is the association of an enlarged seminal vesicle cyst with ipsilateral renal agenesis, which can be symptomatic and require operative interventions.

***Case:*** We present the case of an otherwise healthy 24-year-old man who presented with a symptomatic 15 cm seminal vesicle cyst, which was completely excised using a robot-assisted approach.

***Conclusion:*** Use of robotic surgery for excision of large seminal vesicle cysts is a safe and effective operative procedure.

## Introduction and Background

The mesonephric (Wolffian) duct gives rise to multiple genitourinary organs, including the seminal vesicles, ejaculatory ducts, epididymis, and the vas deferens, along with the trigone of the bladder. Abnormalities from the 4th to 13th week of gestation in the mesonephric duct can lead to ipsilateral failure of ureteral bud proximal growth and association with the metanephric blastema and, in theory, renal agenesis or dysgenesis. Furthermore, ipsilateral abnormalities in the differentiation of the mesonephric duct can also lead to atresia of the ejaculatory ducts, resulting in chronic obstruction of the seminal vesicle and corresponding cystic dilation. The eponym Zinner's syndrome^1^ refers to the triad of renal agenesis, ejaculatory duct obstruction, and seminal vesicle cyst, a rare condition that has an estimated incidence of 0.00035%.^1^

Although these cysts represent benign entities, their management is dictated by the presence of symptoms, including dysuria, frequency, urgency, as well as abdominal, perineal, or scrotal pain. In addition, some patients report pain with ejaculation, recurrent epididymitis, or infertility. Small asymptomatic cysts can be safely monitored for growth.^1^ Larger or symptomatic cysts have been managed with transurethral unroofing, open, laparoscopy, and robot-assisted laparoscopic methods. We report the use of the daVinci robotic platform to facilitate minimally invasive complete en bloc removal of a massively dilated seminal vesicle in a symptomatic patient with ipsilateral renal agenesis.

## Presentation of Case

A 24-year-old male student presented to a community hospital emergency department in September 2016 for a 2-day history of lower abdominal pain, suprapubic pressure, radiating pain to right testicle, subjective fevers, and frequency of urination. He was afebrile, with stable vital signs, and his laboratory work was only notable for a mild leukocytosis to 12,200/μL and a urinalysis with four RBC per high power field. His past medical history was notable for an appendectomy 15 years prior, before start of puberty. He had no notable family history and no children. He underwent a scrotal ultrasonography and a contrast-enhanced CT abdomen and pelvis, which interestingly showed a hypertrophied solitary left kidney and a large cystic mass in the pelvis of unclear etiology. The mass was 11 cm in maximum dimension. He was then transferred to a tertiary care hospital for further evaluation by urology and radiology.

**FIG. 1.**
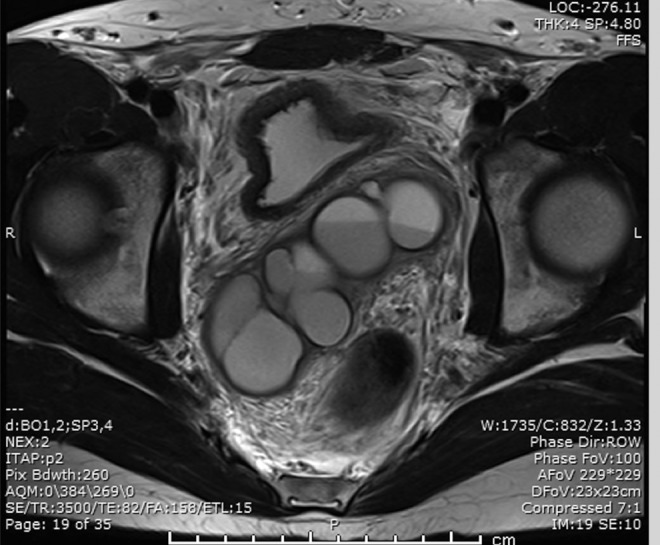
Axial MRI image shows large cystic structure posterior to bladder.

**FIG. 2.**
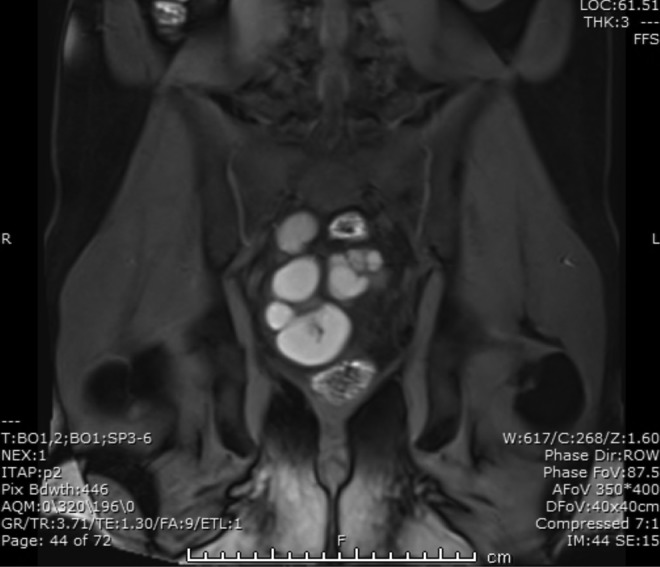
Sagittal view reveals relational anatomy of prostate, bladder, and cyst.

His physical examination was notable for absence of his right vas deferens and for mild suprapubic tenderness to palpation without rebound or guarding. A digital rectal examination was performed, which revealed no abnormalities. He subsequently underwent an MRI with and without IV contrast, to further delineate the anatomy (Figs. 1 and 2). This revealed a large cystic mass consistent with a large right seminal vesicle cyst; the left seminal vesicle was unremarkable. Mass effect from the cyst was seen to be distorting both the prostate and the bladder. A semen analysis was performed, which showed small volume (1 cc) with normal sperm concentration but poor motility and increased abnormal morphology.

After extensive discussion of the risks, benefits, and alternatives the patient was subsequently taken for a robot-assisted laparoscopic removal of the right seminal vesicle cyst. Preceding surgical incision, a cystourethroscopy was performed, which showed a normal anterior and posterior urethra, normal verumontanum, no prostatic utricle, and systematic evaluation of the bladder was notable for absence of the right ureteral orifice, although the trigone itself did not otherwise look atypical.

Abdominal access was performed just superior to the umbilicus with a Veress needle to obtain pneumoperitoneum. Surveillance of the abdomen confirmed the large cystic mass and some minor adhesions in the right lower quadrant from his previous appendectomy. A right vas deferens could not be identified, but the left vas deferens was confirmed to be in orthotopic position. Both deep inguinal rings were closed. Two ports were placed in the patient's left side to assist with taking down of the adhesions, which was performed without issue. Three more ports were placed on the patients' right side: one additional robotic port and two assistant ports. The patient was secured in steep Trendelenburg position and the robot was docked.

**FIG. 3.**
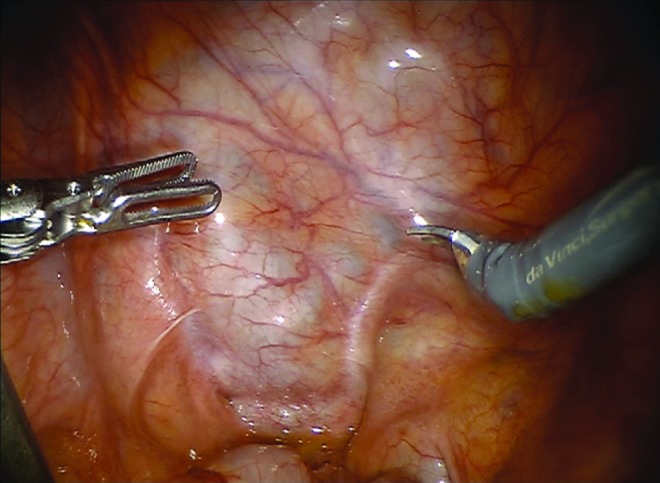
During robot-assisted excision, laparoscopic impression of large cystic structure apparent through peritoneal reflection is evident adjacent to urinary bladder.

The peritoneum was incised to access the seminal vesicle cyst, which was easily identified (Fig. 3). Careful dissection was made from anterior, lateral, and medial attachments. The left vas deferens was identified medially and care was made to dissect it off the cyst without injury. The neck of the cyst was thought to be arising from the pelvic sidewall, but as it was freed, the cyst remained intact. A single 3-0 vicryl figure of eight suture was placed over this area on the pelvic sidewall to reapproximate the excision site; care was made to not include the neurovascular bundle. Excellent hemostasis was maintained. The cyst was then punctured and the fluid was drained to allow for retrieval. An endocatch bag was used to remove the specimen through the initial incision, after widening it superiorly and inferiorly. A 10 flat Jackson-Pratt (JP) drain was placed in the pelvis and the fascia and skin were closed. The operative time was 133 minutes.

The patient had an uneventful postoperative course and was discharged postoperative day 1 after having his JP drain and foley catheter removed. On follow-up, he has had significant improvement in his voiding and abdominal pain symptoms. His erections and ejaculatory status was unchanged postoperatively.

## Discussion and Literature Review

Originally described in 1914,^2^ Zinner's syndrome is a rare condition with fewer than 300 cases reported in the literature. The equivalent syndrome in women has not officially been described, but the obstructed hemivagina and ipsilateral renal abnormality have been implicated in the literature, although this is debatable as it would imply that the embryological origin of the upper vagina would have a Wolffian origin rather than the more traditionally accepted Mullerian origin.^3^

The largest pooled analysis of 52 patients with Zinner's was performed by van den Ouden et al. in 1998.^1^ Their analysis showed that patients can have multiple symptoms, which could come to the attention of a urologist, from lower urinary tract symptoms of obstruction or irritation to nondescript perineal or scrotal pain. A postpubertal male without an ejaculatory duct to drain the seminal vesicle can lead to dilation and swelling of the vesicle until it becomes a cyst large enough to cause these local symptoms. Ouden's review showed that 100% of patients managed with surgical removal of the cyst have improvement in their symptoms, whereas 75% and 30% improve with transurethral unroofing and aspiration, respectively.^1^

**FIG. 4.**
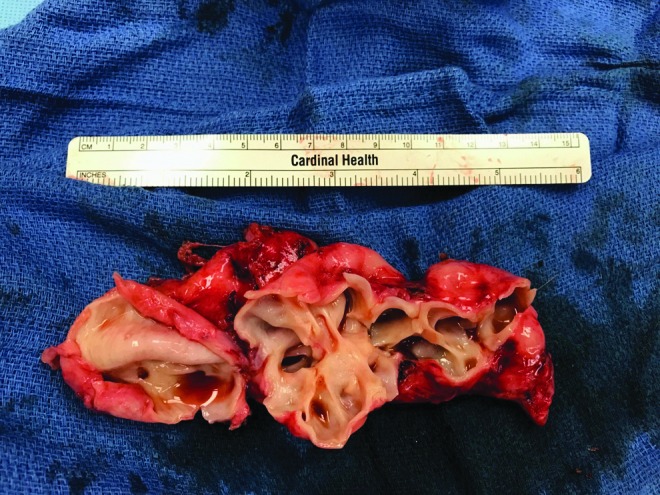
Excised seminal vesicle cyst, bivalved to show hollow cystic structure.

The use of the daVinci robotic surgical device for benign and malignant cases has increased, and previous case reports of robotic excision of these cysts have shown that it can be a safe modality.^4^ The cyst was ∼11 cm in diameter and is the largest reported to our knowledge (Fig. 4). Our patient had a short inpatient stay and a benign postoperative course. Previously, the use of midline abdominal, pfannenstiel, or Gibson incisions for open surgical excision of large cysts were employed, which could possibly have required a longer inpatient stay and a longer recovery time. As with any rare disease, the comfort of the surgeon with the anatomy and the technique should guide management, and referral to a tertiary care center can be beneficial.

## Conclusion

Zinner's syndrome is the rare triad of seminal vesicle cyst, ipsilateral renal agenesis, and ejaculatory duct obstruction, caused by a congenital abnormality of mesonephric development.^1^ Symptoms develop in the second through fourth decade of life as the cyst grows in size and causes local mass effects. Management depends on size, location, symptoms, and comfort of the surgeon. We described the case of a megaseminal vesicle cyst in a patient with Zinner's syndrome removed safely through robot-assisted laparoscopic surgery, the largest cyst reported to have been removed this way.

## Abbreviations Used

CT: computed tomography
MRI: magnetic resonance imaging
